# The role of medullary astrocytes in breathing and arousal: insights into glial regulation of respiratory function

**DOI:** 10.21203/rs.3.rs-6063056/v1

**Published:** 2025-03-13

**Authors:** Jan Marino Ramirez, Luiz Oliveira, Nicole Miranda, Hyun-Kyoung Lim, Liza Severs, Ana Takakura, Thiago Moreira, Franck Kalume

**Affiliations:** Seattle Children’s Research Institute; Universidade de São Paulo; Seattle Children’s Research Center; Hyun-Kyoung; Seattle Children’s Research Institute, The University of Wasington; Universidade de São Paulo; University of São Paulo; Seattle Children’s Research Institute

**Keywords:** purinergic, preBötzinger complex, sigh, central chemoreception

## Abstract

Astrocytes play vital roles in regulating brain states across organisms. Specifically, they serve several roles in regulating breathing behaviors and associated brain states, including facilitating transitions between phases of breathing by sensing small changes in O_2_ and CO_2_ levels, regulating the sleep-wake cycle, and impacting arousal and wakefulness. Here, we tested the hypothesis that astrocytes in the ventral respiratory column (VRC) are important for arousal and sigh generation in alert mice (Aldh1l1^Cre^). Using calcium imaging we show that some Aldh1l1 cells are phase-locked with sigh generation and are activated in the VRC by hypoxia. Optogenetic activation (AAV-CAG-ChR2-EYFP) of astrocytes in the VRC increased the probability of evoking sighs and arousal while awake and during non-rapid eye movement (NREM) sleep. Depletion of astrocytes in the VRC by an AAV-CAG-Caspase3 virus (ablation of 77%) does not impact the probability of sigh generation in any sleep-wake state under control conditions. However following the depletion of astrocytes, arousal and sigh generation is significantly delayed in response to hypoxia (65.3 ± 5.5 vs. control: 21.7 ± 1.9 seconds). We conclude that medullary astrocytes play a critical role in the generation of arousal and sighs particularly in response to hypoxia.

## Introduction

Breathing is vital for sustaining life, as it ensures the continuous exchange of oxygen (O_2_) and carbon dioxide (CO_2_) in the body ^1,2^. This process requires precise, uninterrupted regulation to meet the metabolic demands of the organism ^1,2^. This control relies on a brainstem network of specialized neurons and astrocytes responsible for orchestrating the responsiveness, as well as the rhythm and pattern of respiration ^3–6^. A critical structure within this network is the ventral respiratory column (VRC), which contains key regions such as the pre-Bötzinger Complex (preBötC) ^7,8^. This area serves as the primary rhythm generator for breathing, that maintains a steady inspiratory cycle ^1,9^. Through intricate coordination, these neuronal circuits adjust breathing patterns in response to changing physiological needs, ensuring that the body maintains proper levels of O_2_ intake and CO_2_ elimination ^8–10^.

Astrocytes play an essential role in preserving network stability by regulating neurotransmitter concentrations, maintaining ionic balance, and influencing synaptic transmission ^11–14^. Astrocyte function extends beyond merely supporting neurons; astrocytes are actively involved in managing transitions between different breathing phases and responding to subtle fluctuations in O_2_ and CO_2_ levels, which is critical for sustaining consistent homeostasis ^6,11,15,16^. Furthermore, astrocytes are key players in regulating the sleep-wake cycle, directly influencing states of arousal and alertness ^14,17,18^. By releasing gliotransmitters, modulating neuronal activity, and maintaining the integrity of the blood-brain barrier, these glia cells help to preserve cognition, and other behavioral functions during wakefulness and sleep ^14,15,19,20^.

Recent studies revealed that astrocytes play a critical role in generating sighs through the release of adenosine triphosphate (ATP) ^11,14,21^, which involves the activation of P2Y1 receptors in the preBötC as shown *in vitro* ^21^. Sighs constitute a unique respiratory pattern that serves both physiological and emotional purposes ^22–24^. It is thought to act as a reset mechanism for the respiratory system, providing a deep breath that helps maintain lung function by preventing alveoli from collapsing ^22,25,26^. Emotionally, sighing often reflects shifts in arousal states, marking transitions such as moving from stress to relaxation or signaling moments of anticipation or relief ^26^. This study delves into the broader role of astrocytes in modulating both breathing, sighing and arousal states transitions.

## Results

In a cohort of 7 freely behaving mice (Supplementary Fig. 1), the sigh frequency increased while animals were sleeping in NREMS (0.98 ± 0.02 sighs/min) or REMS (1.36 ± 0.15 sighs/min) compared to the awake state (0.55 ± 0.03 sighs/min; p = 0.014; one-way ANOVA; F_(2,20)_ = 19.45). Confirmed by our recordings, we also found a high probability of sighing and arousal occurring together (0.87; Supplementary Fig. 2).

### Astrocytes opto-stimulation induce arousal and sigh

**Figure 1:**
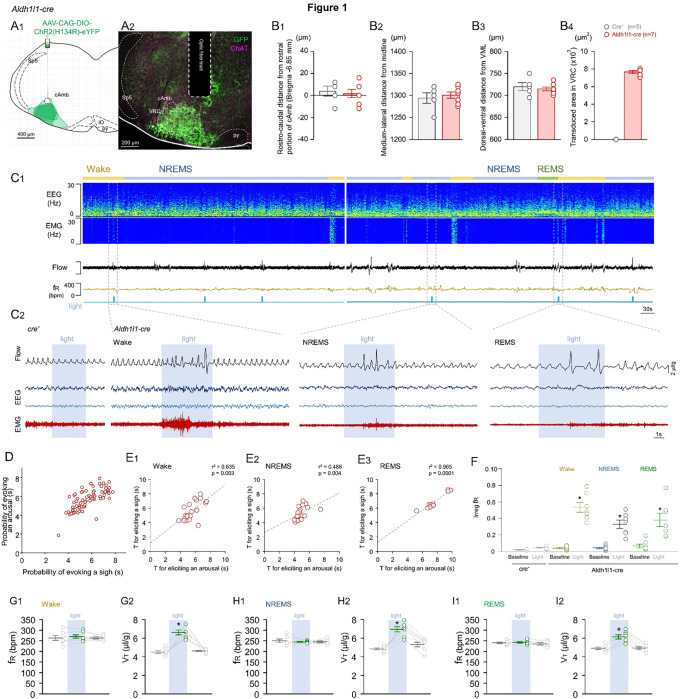
Photo-stimulation of Aldh1l1 astrocytes in the VRC elicited arousal with sighs. A) Representative A1) Plots and A2) Image showing the localization of AAV-CAG-ChR2 transduction and fiber optic placement in the VRC. B) Anatomical location of the fiber optic tip in relation to B1) Rostro-caudal distance from rostral portion of cAmb; B2) Medium-lateral distance from midline; B3) Dorsal-ventral distance from the VML; and B4) Represents the transduced area reached by viral injections into VRC of Aldh1l1^Cre^ mice. C) Representative records of EEG and EMG waves and respiratory flow in freely behaving mice during the opto-activation. C2) Shows extended traces of these opto-stimulation generating arousal with sighs. D) Pierson correlation of all the episodes of arousal in wake, NREM, and REM after Aldh1l1 opto-stimulation and E) separated Pierson correlation when animals were E1) awake; E2) in NREM; and E3) in REMS. F) Regularity score. G-I) Respiration changes during opto-stimulation of Aldh1l1. No change was observed in f_R_ for Wake, NREM, and REMS (G1, H1, I1), but V_T_ increased in all states (G2, H2, I2) Abbreviations: cAmb, nucleus ambiguus pars compact; VRC, ventral respiratory column; py, pyramids. Sp5, trigeminal nucleus, IO: inferior olives. Statistical analysis: One-way ANOVA (*statistically significant from baseline).

To manipulate astrocyte activity, we used Aldh1l1^Cre^ adult mice that received injections of AAV-CAG-ChR2 into the VRC, followed by inserting fiber optic and EEG/EMG electrode implants (Fig. 1). To ensure accurate placement, we assessed the precise location of the fiber optic implants based on medio-lateral (1,301 ± 12 μm), dorso-ventral (714 ± 8 μm from VMS), and rostro-caudal axes (4 ± 4 μm from the rostral portion of cAmb) (Fig. 1B). Due to the diffuse and complex morphology of astrocytes, we measured the transduced area by injecting the virus into the VRC. In the Aldh1l1^Cre^ mice used in our experiments we found transduction in an area of 7,677 ± 150 μm^2^, divided across three brain sections (bregma levels −6.75 to −6.85 mm), extending dorso-ventrally from the compact portion of the ambiguus nucleus (cAmb) to the marginal layer of the ventrolateral medulla (Fig. 1A–B).

Optogenetic activation of Aldh1l1-transduced astrocytes leads to an increased EMG neck tone, a change in respiratory pattern, and the induction of sighs in both awake and sleeping animals. Figure 1C depicts representative EEG/EMG recordings together with the normalized (by resting breathing) plethysmography data and optogenetic stimulation. As demonstrated in the representative recordings we found in some cases two consecutive sighs, each followed by the characteristic post-sigh apnea (Fig. 1C2). In contrast, in cre^(−/−)^ AAV-CAG-ChR2-eYFP-injected mice, no sighs or arousal were evoked in any wake-sleep state. The probability of evoking an arousal versus a sigh in this cre^(−/−)^ group was similar to the probability of generating spontaneous sighs (not shown). Across all wake-sleep states there was a high correlation of eliciting sighs and arousals together. The averaged time for eliciting a sigh and arousal was 5.54 ± 0.23 seconds while the animals were awake and 4.93 ± 0.19 seconds while animals were in NREMS (Fig. 1E). However, as shown in Fig. 1E3, transitioning from REMS to arousal required a more prolonged stimulation compared to other states (7.66 ± 0.46 seconds; p = 0.045; one-way ANOVA; F_(2,46)_ = 15.24).

Astrocyte activation in the VRC did not affect the eupneic f_R_, but V_T_ of eupneic activity was increased in wakefulness (6.60 ± 0.27 vs. before: 4.48 ± 0.15 μl/g; p < 0.001; one-way ANOVA; F_(2,20)_ = 37.86), NREMS (6.94 ± 0.29 vs. before: 4.48 ± 0.10 μl/g; p < 0.001; one-way ANOVA; F_(2,20)_ = 34.31) and REMS (6.12 ± 0.23 vs. before: 4.90 ± 0.14 μl/g; p < 0.001; one-way ANOVA; F_(2,20)_ = 16.07) (Fig. 1 and Table 1–2). Breathing frequency also became more irregular when optogenetically activating astrocytes in all wake-sleep states (Fig. 1F). In this analysis (Fig. 1F–I), we excluded sigh events, so the data represents only eupneic breathing during astrocyte optogenetic activation.

**Figure 2:**
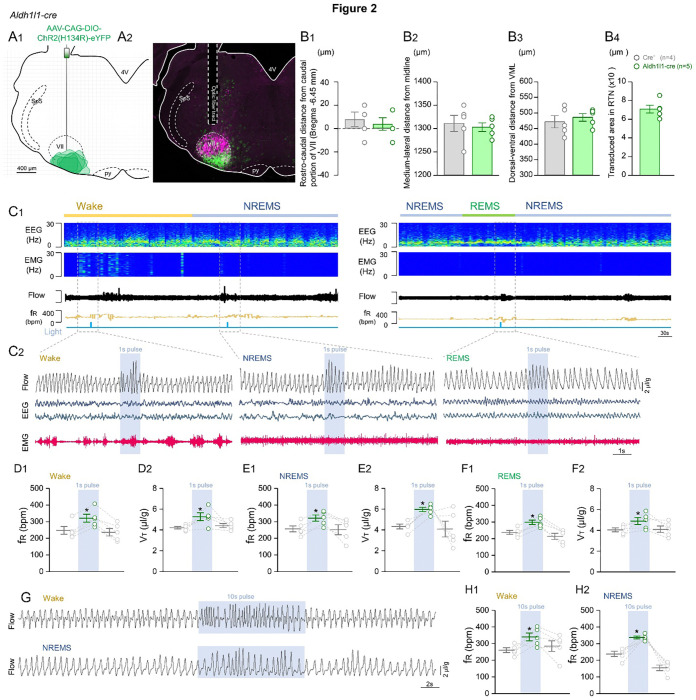
Photo-stimulation of Aldh1l1 astrocytes in the RTN elicited increased respiratory parameters, without arousal with sighs. A) Representative images showing A1) Plots and A2) Image, showing the localization of AAV-CAG-ChR2 transduction and fiber optic placement in the RTN. B) Anatomical location of the fiber optic tip in relation to B1) Rostro-caudal distance from caudal portion of VII; B2) Medial-lateral distance from midline; B3) Dorsal-ventral distance from the VML; and B4) Represents the transduced area reached by our viral injections into RTN of Aldh1l1^Cre^ mice. C) Representative recordings of EEG and EMG waves and respiratory flow in freely behaving mice during the 1 second of opto-activation. C2) Shows extended traces of opto-stimulation generating arousals and sighs. Respiration changes during opto-stimulation of Aldh1l1 cells in 1) f_R_ and 2) V_T_ in D) Wake; E) NREMS; and F) REMS. G) Representative recordings of respiratory flow in freely behaving mice during 10 second opto-activation. Graphs of f_R_ changes during 10 seconds of opto-stimulation of Aldh1l1 cells while H1) awake and H2) NREMS. Abbreviations: VII, facial nucleus; VRC; py, pyramids. Sp5, trigeminal nucleus; 4V, fourth ventricle. Statistical analysis: One- or Two-way ANOVA (*statistically significant from baseline).

We next transduced an area of 7,080 ± 418 μm^2^ by placing the injections and fiber optics slightly rostral compared to the preBötC region in order to target the RTN, the main chemosensitive region in the CNS. Unlike in the VRC, optogenetic activation of astrocytes in the RTN increased both the eupneic f_R_ and V_T_ while the mice were awake, or during NREM and REM sleep (Fig. 2), without evoking sighs or arousal. Even during 10-second stimulation trials, we did not observe the presence of these events (Fig. 2G). We also found higher irregularity during optogenetic stimulation (Data not shown).

### Depletion of astrocytes in the VRC does not abolish sigh and arousal generation

**Figure 3:**
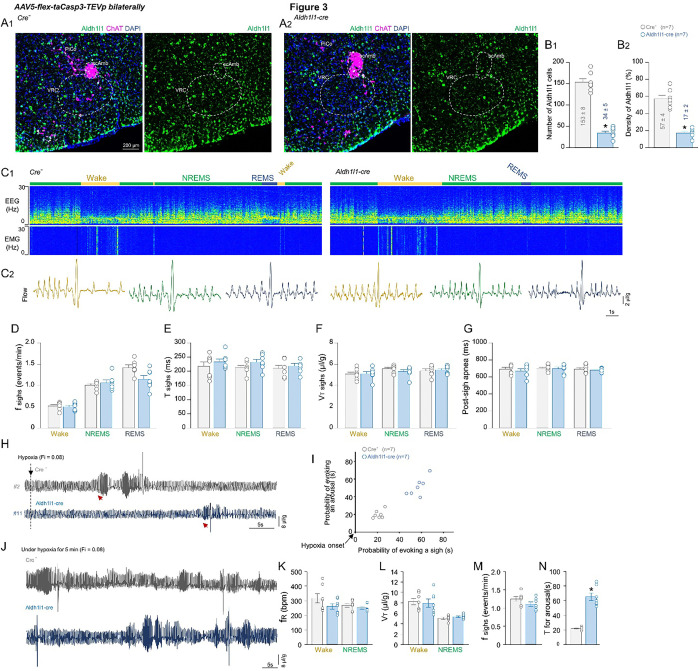
Depletion of Aldh1l1 astrocytes in the VRC does not impact spontaneous arousal and sighs. A) Representative images showing the expression of Aldh1l1 (green), ChAT (magenta) and DAPI (blue) in (A1) cre^(−/−)^ and (A2) Aldh1l1^Cre^ mice injected bilaterally with AAV-CAG-taCasp3. B) Mean of B1) Number and B2) Density of Aldh1l1 positive cells and quantified in the VRC. C1) Representative records of EEG and EMG waves and respiratory flow, C2) Detail for sigh events in freely behaving mice in control or after depletion of Aldh1l1 cells into VRC. Respiratory parameters D) frequency E) Time and F) V_T_ of sighs and G) post-sigh apnea duration were not affected by astrocyte depletion. H) Respiratory flow recordings demonstrating the onset of hypoxia and I) the probability of evoking an arousal/sigh in control or Aldh1l1-lesioned animals. J) Respiratory recordings after 5 min of hypoxia. Mean of K) f_R_ and L) V_T_ parameters; M) frequency of sighs and N) Time for arousal in cre^(−/−)^ and Aldh1l1^Cre^ mouse after AAV-CAG-taCasp3 injection into VRC. Abbreviations: scAmb, nucleus ambiguus pars semi-compact; VRC, ventral respiratory column; PiCo, postinspiratory complex. Statistical analysis: One- or Two-way ANOVA (*statistically significant from baseline).

To investigate whether spontaneous arousal and sighs would still occur in freely behaving mice after regional astrocyte depletion, we recorded respiration and EEG/EMG data in Aldh1l1^Cre^ or cre^(−/−)^ mice after bilaterally injecting AAV9-CAG-DIO-taCaspase3-TEVp-WPRE into the VRC (Fig. 3). Following these experiments, we observed a depletion of approximately 77% of astrocytes in Aldh1l1^Cre^ mice (34.1 ± 4.5 vs. cre^(−/−)^: 153.1 ± 8.3 cells; p < 0.001; t-test; t_(1,13)_ = 8.841). Despite this depletion, when analyzing the functional data over ~ 5 hours, we still observed sigh and arousal episodes in the astrocyte-depleted group. The frequency and amplitude of sighs were not significantly different from the cre^(−/−)^ group (Fig. 3D–G).

Astrocytes in the preBötC are known to detect changes in oxygen levels. To test this, we exposed all animals to hypoxia (10% O_2_) for one hour and evaluated the time needed to induce arousal/sighs. VRC astrocyte depletion affected the acute phase of the hypoxic response which occurs shortly after oxygen levels decrease and consists of the arousal and an increased ventilation (as shown in Fig. 3). The group with astrocyte depletion in the VRC had a delayed arousal response to hypoxia (65.25 ± 5.09 vs. cre^(−/−)^ : 21.77 ± 0.87 s; p = 0.002; t-test; t_(1,13)_ = 28.0, Fig. 3H). After 50–60 minutes of hypoxia, we did not observe significant changes in f_R_ and V_T_ between the two groups. The frequency of sighs in the Aldh1l1-depleted group was not statistically altered compared to the cre^(−/−)^ group (Aldh1l1^Cre^: 1.11 ± 0.06 vs. cre^(−/−)^ : 1.24 ± 0.06 sighs/min; p = 0.133; t-test; t_(1,13)_ = 1.612, Fig. 3J–K).

### Interaction of astrocytes and C1 neurons may be important for arousal and sigh generation

**Figure 4:**
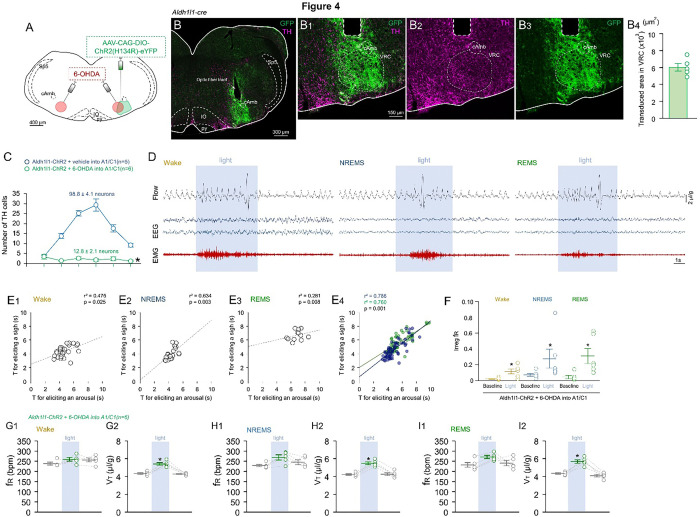
Photo-stimulation of Aldh1l1 astrocytes in the VRC elicited arousal with sighs, with and without A1/C1 toxin depletion. A) Schematic protocol. B) Representative image showing the localization of B1) AAV-CAG-ChR2 transduction and fiber optic placement in the VRC, B2) TH staining (magenta) and B3) AAV-CAG-ChR2 (green). B4) Representation of area of VRC transduced by viral injections in Aldh1l1^Cre^ mice; C) Rostro-caudal distribution of TH neurons along bregma levels. D) Representative records of EEG and EMG waves and respiratory flow in freely behaving mice during the opto-activation generating arousal with sighs. E) Pierson correlation between T for sigh and arousal when animals were E1) wake; E2) NREM and E3) REMS in the lesioned-group with E4) comparison of all arousal episodes after Aldh1l1 opto-stimulation in intact (blue) or TH-lesioned (green) animals. F) Regularity score. G-I) Respiration changes observed during opto-stimulation of Aldh1l1 cells in the absence of TH-A1/C1 neurons in Wake (G1/2) No change was observed in G1) f_R_ but stimulation increased V_T_ (G2). F_R_ was not increased during H) NREMS and I) REMS, but did alter V_T_ for both NREMS and REMS states. Abbreviations: cAmb, nucleus ambiguus pars compact; VRC, ventral respiratory column; py, pyramids. Sp5, trigeminal nucleus; IO, inferior olives. Statistical analysis: One- or Two-way ANOVA (*statistically significant from baseline).

It was elegantly reported by Burke and colleagues that C1-noradrenergic neurons, located within the VRC and the caudal portion of the RTN, are crucial for arousal and sigh generation ^27^. Based on this, we hypothesized that the interaction between astrocytes and C1 neurons could play a role in eliciting arousal and sighs in mice. To test this hypothesis, we conducted the same Aldh1l1 photo-stimulation in the VRC as presented in Fig. 1, but in addition to ChR2-CAG-AAV and fiber optics, we also injected the toxin 6-OHDA bilaterally targeting C1/A1 neurons in the ventrolateral medulla (VLM). This toxin, a dopamine analogue prone to oxidation, is taken up by the dopamine transporter, enabling selective damage to catecholaminergic neurons. As shown in Fig. 4, the bilateral 6-OHDA injections into the C1/A1 regions resulted in a depletion of ~ 87% of tyrosine hydroxylase (TH)-positive cells in the VLM (12.8 ± 2.1 vs. vehicle: 98.8 ± 4.1 TH^+^ neurons; p < 0.001; t-test; t_(1,10)_ = 18.77). Similar to the data in Fig. 1, the AAV-CAG-ChR2-eYFP transduced an area of 6,028 ± 447 μm^2^ in the VRC, spreading ± 50 μm rostro-caudally (Fig. 4B4).

Despite this significant depletion of TH-positive C1/A1 neurons, Aldh1l1 photo-stimulation in the VRC still elicited sigh and arousal responses (Fig. 4D). When compared to vehicle-C1/A1 injected animals, the time required to trigger both sighs and arousal was slightly but significantly delayed in the 6-OHDA-C1/A1 injected mice (5.75 ± 1.46 vs. vehicle: 5.08 ± 1.28 seconds; p = 0.003; t-test; t_(1,107)_ = 3,099, Fig. 4E). This suggests that the interaction between astrocytes and C1 neurons may contribute to the rapid sigh and arousal responses. This could be achieved by exerting neuromodulatory effects on C1/A1 neuronal activity.

Similar to Fig. 1 data, optogenetic activation of astrocytes in the VRC increased the V_T_ and also increased the f_R_ irregularity in all the wake-sleep states (Fig. 1F–I).

### Aldh1l1 cells in the VRC are associated with sigh generation and activated during hypoxia

To investigate whether astrocytes could be activated by hypoxic or anoxic challenges, we continued using Aldh1l1^Cre^ mice. Adult mice were acclimated to the plethysmography chamber for two hours, after which they were exposed to either one hour of hypoxia (10% O_2_) or a brief episode of anoxia (100% N_2_) lasting no more than 30 seconds. Room air was immediately restored once animals lost consciousness to allow full recovery. Thirty minutes after the end of both stimuli, the animals were deeply anesthetized and perfused.

**Figure 5:**
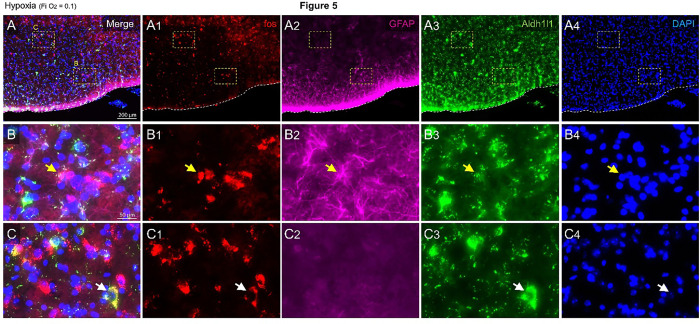
Hypoxia- induced Aldh1l1 cells-activated in the VRC. Representative photomicrographs of A) VRC demonstrating and higher magnification in B-C. 1) Fos (red), 2) GFAP (magenta), 3) Aldh1l1 (green) and 4) DAPI (blue) expression while the animals were exposed to hypoxia (Check protocol for details).Yellow arrow point the Aldh1l1/GFAP/Fos positive astrocyte and white arrow point the Aldh1l1/Fos positive/ GFAP negative cell. Scale in B applies to B-C.

Hypoxia and anoxia led to the activation of Aldh1l1 cells in the VRC as measured by an increase in astrocyte Fos expression (Hypoxia: 63.5 ± 5.6; Anoxia: 77.7 ± 5.2 vs. Normoxia: 4.2 ± 1.2 Fos^+^/GFAP^+^/Aldh1l1^+^ cells; p = 002; one-way ANOVA; H_(2,11)_ = 8.346). We also report the presence of cellular activation in the NTS, LC, PBN, and KF during hypoxia and anoxia (Fig. 5 and Supplementaries 3 and 4).

**Figure 6:**
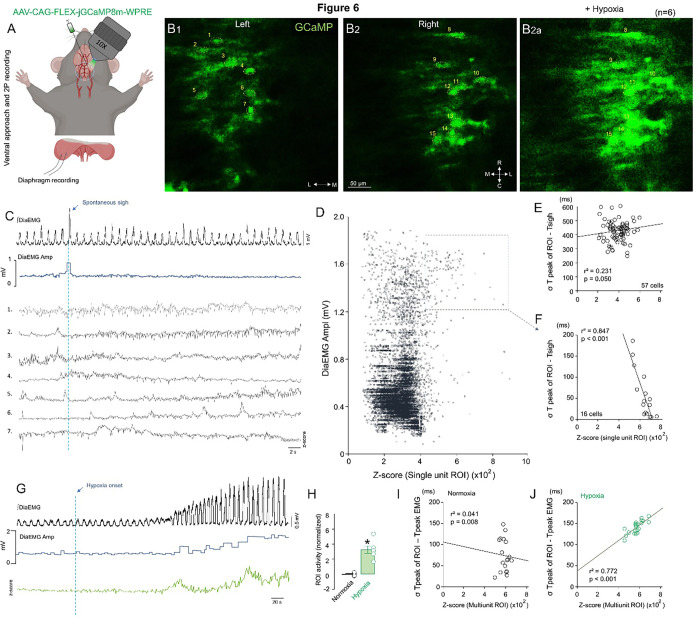
Calcium transient waves of Aldh1l1 cells in baseline or hypoxia. A) Schematic of calcium imaging ventral approach. B) Representative screenshot of the GCaMP expression in the VRC after AAV-CAG-GCaMP injection into VRC at baseline. 2A shows the maximum peak of activity during hypoxia. C) Diaphragm EMG and he related amplitude paired with unicellular ROIs at baseline showing a spontaneous sigh. D) Plots of each ROI (600 samples/run) vs. Dia EMG amplitude. E-F) Correlation of ROIs vs. temporal difference between peak of ROI and sigh onset in E) non-phase locked and F) phase-locked cells. G) ROI activity in baseline and hypoxia shows an increased Aldh1l1 positive cells activity when respiration increases amplitude. H) Normalized graph of multiunitary Ca^+^ imaging recording under normoxia and hypoxia. Correlation of multiunitary ROIs vs. temporal difference between peak of ROI and peak of Dia amplitude shows no correlation under I) normoxia and correlation under J) hypoxia. Statistical analysis: One-way ANOVA (*statistically significant from baseline).

In another approach, Aldh1l1^Cre^ mice received AAV-CAG-GCaMP injections into the VRC. Twenty-one days later, the animals were sedated with urethane ip., and the VMS was exposed while recording diaphragm EMG, as demonstrated in Fig. 6. Calcium imaging data revealed that some rhythmogenic preBötC astrocytes in the VRC (22% of recorded cells, 16 out of 72 cells) were rhythmically phase-locked to sigh generation (Fig. 6D and F). This group of cells was analyzed together, and we found a high correlation between each ROI and the delay of the ROI signal relative to sigh generation (r^2^ = 0.847). We also measured the correlation between non-phase-locked astrocytes activity and sigh generation (57 out of 72 cells) and found a low, non-significant correlation. However, these astrocytes exhibited occasional high ROI activity randomly, and the probability of a non-phase-locked astrocyte lighting up during eupnea burst was 0.103.

We also measured the activity of these cells under hypoxia for 30 seconds. As shown in Fig. 6G, calcium imaging revealed that Aldh1l1-positive cells displayed increased activity during hypoxia, and the peak activity measured in the ROIs correlated highly with the peak of the Dia EMG signal, differing from normoxia data (Fig. 6I–J). Taken together, these data suggest that this population of cells may be important for central chemoreception in response to low oxygen levels and may play a role in sigh generation.

## Discussion

Here we explored the role of astrocytes in the generation of sighs and arousal in freely behaving mice, with a specific focus on the astrocyte populations in the VRC and RTN. Our study builds on prior studies that demonstrate that astrocytes play a crucial role in regulating breathing, arousal, and sigh generation ^5,6,11,21,28,29^. These studies showed that astrocytes influence rhythmogenic neurons in the preBötC via purinergic signaling ^30,31^. Sighing, a distinct type of deep breath that is generated by excitatory neurons in the preBötC involves various sigh-specific cellular mechanisms ^21,25,32–35^. In freely behaving mice, we measured respiratory patterns along with EEG and EMG recordings and found that astrocytic activation in the preBötC increased the V_T_ and induced sighs and arousal, especially during sleep states like NREMS and REMS. Arousal was also reflected in increased muscle tone. In this study, we show that sighs were consistently followed by an arousal, as has previously been described ^36,37^.

By contrast, astrocytic stimulation in the RTN, evoked neither sighs nor arousal, though it affected eupneic breathing by increasing f_R_ and V_T_. The effect on eupneic breathing is consistent with the known role of the RTN as an important chemosensitive region. The RTN plays a critical role in detecting changes in CO_2_ levels in the blood and cerebrospinal fluid, which translates into pH changes ^38–41^. Astrocytes in the RTN have been shown to play a crucial role in central chemoreception by detecting decreases in pH and releasing ATP, which then stimulate nearby RTN neurons, and further act as a vasoconstrictor in the region ^15,38,40^. This signaling amplifies the respiratory response, enhancing the drive to breathe more rapidly and expel excess CO_2_^38,42^. Neuronal activation of the RTN speeds up the f_R_ and also provokes sleep-wake cycle transitions or arousals ^43–45^. Thus, astrocytes act as intermediaries, boosting the sensitivity of RTN neurons to CO_2_ and fine-tuning the chemoreceptive process to maintain blood pH and overall respiratory homeostasis ^46^. However, our data suggest that astrocyte activation in the RTN does not play a critical role in the induction of arousal and the generation of sighs. The differential effects observed for RTN and preBötC are consistent with the concept that astrocytes in different brain regions play differential roles in the central control of breathing, central chemosensation, arousal and sleep/wake transitions. This is also consistent with the notion that astrocytes do not function only as simple support cells for neurons, helping with metabolic and structural support ^14,20,47^, but rather that astrocytes are active participants in regulating rhythmic behaviors, chemoreception, arousal and in the sleep/wake cycle ^17,48^.

Since a concerted activation of arousal and sighs was only observed when stimulating the VRC, specifically the region of the preBötC, we hypothesized that astrocytes located in the VRC interact with catecholaminergic C1 neurons. It has previously been demonstrated that optogenetic stimulation of C1 neurons induces arousal with sigh events in rats ^27^. Here we show that after depleting C1 neurons with 6-OHDA, sighs and arousals were still elicited following optogenetic stimulation of astrocytes. However, the time to evoke these responses was delayed compared to vehicle-injected animals. Based on these findings we propose that C1 neurons are part of the arousal circuitry ^27,49^, but they are not necessary for arousal and sigh generation. However, astrocytes and C1 neurons together likely enhance the generation of arousal and sights.

Following a significant depletion of astrocytes (around 77%) in the VRC, spontaneous sighs and arousal episodes persisted at levels comparable to those observed in non-lesioned controls. Interestingly, we also report in our study the presence of Aldh1l1 cells in the VRC exhibiting rhythmic activity phase-locked with sighs. Even though we found a significant number of these cells, a portion of astrocytes in the preBötC are not directly rhythmogenic or related to sighs or even eupnea. However, we can confirm their potential involvement in sigh modulation, as previously demonstrated ^21^. We also found that this population of astrocytes in the VRC increased their activity under hypoxic conditions. This finding supports the idea that astrocytes contribute to the brain’s detection of low oxygen levels and likely play a role in generating corrective respiratory behaviors like sighing ^6,11,21^.

Here we show that in response to hypoxia (10% O_2_), astrocyte depletion in the VRC significantly delayed the onset of both sighs and arousal. The delay could be physiologically significant given that a rapid arousal response is critical when facing hypoxic conditions. This supports the notion that disturbances in sighing and arousal could contribute to the failure to arouse in sudden infant death syndrome (SIDS) ^50^. This delay is also consistent with the finding that SIDS victims show gliosis in the area of the preBötC ^51^. Thus, central medullary astrocytes play an important role in oxygen sensing and in modulating adaptive responses to low oxygen environments ^6,15,30^. This is also supported by the finding that astrocytes in the VRC are activated in response to hypoxia and anoxia ^6^. Here we showed hypoxia and anoxia induced Fos expression in astrocytes and neurons in several areas of the brainstem. Thus, our data illustrate a broader role for astrocytes and neurons in coordinating the hypoxic response across multiple brain regions.

Overall, these results suggest that while astrocytes in the VRC contribute to respiratory modulation, arousal, and sigh generation, they are part of a broader network involving other neuronal populations, and potentially modulate neural dynamics in neuronal populations including C1 region and the preBötC. This interaction between astrocytes and neurons in the VRC and surrounding regions appears to play a crucial role in maintaining respiratory homeostasis, controlling the rhythmicity responsible for adapting to environmental changes.

## Methods

Experiments were performed on 63 adult C57BL6/J mice weighing between 25–30g. All experimental and surgical procedures were conducted in accordance with the National Institutes of Health and to the defined guidelines by the Seattle Children’s Research Institute (SCRI) animal care and use committee (Protocol Number: 00033). Aldh1l1^Cre^ mice (Hemizygous) were obtained from Jackson Laboratories [FVB-Tg (Aldh1l1^Cre^)^JD1884Htz/J^, Jax stock No. 023748], and were bred at the Seattle Children’s Research Institute animal facility. The animals had free access to water and food and were housed in a temperature-controlled (22 ± 1°C) facility with a 12:12 h light/dark cycle.

### Surgical procedures

#### Adenovirus Injections

Aldh1l1^Cre^ animals were anesthetized with isoflurane (2.0–2.5% balanced with 100% oxygen) and fixed in a stereotaxic apparatus (Stoelting model 620), followed by local administration of lidocaine (7 mg/kg), and a small craniotomy using a micro drill (0.7 mm). After cranial opening, unilateral injection of AAV-CAG-DIO-ChR2(H134R)-eYFP [AAV PHP.eB, 2.1×10^13^ GC/ml, Cat # 127090-PHPeB, Gift from Viviana Gradinaru ^52^], AAV-CAG-FLEX-jGCaMP8m-WPRE (AAV1, 2.2×10^13^ GC/ml, Cat # 162381-AAV1), both acquired from Addgene; or bilateral injections of AAV-CAG-DIO-taCaspase3-TEVp-WPRE (AAV9, 7×10^12^ GC/ml, Cat # NTA-2012-ZP273), acquired from Creative Biolabs, were administered (50 nL/injection) using a glass micropipette attached to the Nanoject equipment (Thomas scientific). Injections targeted the VRC (focusing on preBötC region) with the following coordinates: 1.7 mm caudal to the lambda, 1.2 mm lateral to the midline, and 4.5 mm ventral to the dura mater; or retrotrapezoid nucleus (RTN): 1.6 mm caudal to the lambda, 1.3 mm lateral to the midline, and 4.7 mm ventral to the dura mater ^53^.

Following AAV-CAG-ChR2-eYFP viral injections: Fifteen days later, mice were implanted unilaterally with one 125 μm fiber optics (RWC, Cat # 807–00022-00) targeting the preBötC or RTN, using a 15-degree rostral to caudal sagittal angle. We used the following coordinates, a) preBötC: 1.1 mm caudal to the lambda, 1.2 mm lateral to the midline, and 4.8 mm ventral to the dura mater; or b) RTN: 1.0 mm caudal to the lambda, 1.3 mm lateral to the midline, and 5.0 mm ventral to the dura mater. For freely behaving experiments, all the animals (AAV-CAG-Ch2R-eYFP and AAV-CAG-Casp3) also received implantation of plate screws in the parietal bone region, and received electroencephalogram (EEG) and electromyogram (EMG) electrodes for EEG recordings and EMG recording of the neck muscle. Additionally, a reference electrode was placed in the occipital bone region to minimize wave noise, and a ground electrode was placed subcutaneously in the anterior chest region.

After all surgical procedures, fiber optics and electrodes were fixed using dental cement (Embrace wetbond, Pulpdent Corporation, Cat # 231215) and mice were placed in individual cages and treated with Ketofen (7 mg/kg/day for 2 days), monitored daily, and maintained 5–6 days for acclimatation and functional experiments. The AAVs, fiber optics or electrodes did not produce any readily observable behavioral effects.

#### Ventilation and EEG/EMG recordings

Twenty-one days after AAVs injections, mice were briefly anesthetized with isoflurane (5%, < 30 s), carefully tethered to the fiber optic cable connected to a blue (447 nm) laser and DPSS driver, and connected to the EEG/EMG cables (at 7 a.m.) and allowed at minimum 2 hours to acclimate to the chamber environment. Recordings began after acclimatization (at 9 a.m.). Three consecutive days prior to experimental procedures, animals were acclimatized for one hour/day.

##### EEG/EMG:

Continuous recordings were obtained via a slip ring (Air Precision, Le Pressis Robinson) that enabled the mice to move freely. The signals were amplified by 10,000X (CyberAmp 380, Axon Instruments, California, USA), with EEG signals filtered between 1–70 Hz and EMG signals between 10–300 Hz. The data were sampled at 1 kHz per channel and recorded using a Cambridge digitizer (micro1401, CED, Cambridge, UK) and Spike 2 software (version 7.07, CED). EEG and EMG data was classified as wakefulness, rapid eye movement sleep (REMS) or non-rapid eye movement sleep (NREMS) based on the following criteria: Wakefulness was identified by sustained muscle activity in the EMG with EEG patterns showing low-amplitude, high-frequency waves; NREMS was marked by a decline in muscle tone and exhibited medium-amplitude, low-frequency waves in EEG readings; and REMS was distinguished by muscle atonia and EEG patterns displaying low-amplitude, high-frequency activity. In addition to the EEG/EMG recordings, we also confirmed the sleep-wake states by video recording the animals throughout the experiments.

###### Ventilation

Pulmonary ventilation (V_E_, μl/g/min) measurements were conducted as outlined by Miranda et al., 2024 ^54^. In summary, respiratory rate (f_R_, respiratory frequency) and tidal volume (V_T_, μl/g) were assessed using whole-body plethysmography. This method involved a sealed and constantly ventilated chamber with 1–2 L/min of air (21% oxygen balanced with nitrogen), maintaining stable temperature and humidity (21 ± 0.5°C and ± 10%, respectively). Pressure transducers recorded small changes in pressure inside the chamber to obtain various respiratory cycle parameters. We also calculated the regulatiry score as the ratio of the standard deviation (SD) to the mean of f_R_^53^. Calibration of volume was performed for each ventilation measurement by injecting a calibrated air volume (1 mL) into the chamber. Flow controllers were adjusted for hypoxia (8% O_2_ balanced with N_2_) challenges. The pressure signals were amplified, filtered, and recorded for offline analysis using PowerLab software (PowerLab 16/30, ML880/P; ADInstruments, New Zealand) ^53^.

A sigh was identified when the inspiratory peak increased by twice the amplitude of a eupneic breath and followed by an apnea (Supplementary 1). EEG and EMG recordings were conducted within the plethysmography chamber for 6 continuous hours, starting at 9 o’clock in the morning. Offline analysis of the sleep-wake cycle and ventilation were performed in a blinded manner using LabChart8 software.

#### Optogenetic stimulations

Once the animals showed calm behavior and a continuous stable respiratory rate, we recorded ventilation and EEG/EMG for 30 minutes. After this baseline period (no stimulation), optogenetic stimulations were performed by the experimenter randomly using a 7–8 mW laser power. The stimulations were performed for 5–10 seconds every 30 minutes. We attempted multiple stimulations per day of experiments to obtain events where animals were awake or sleeping in REMS and NREMS. Later, we were only able to distinguish those moments in our offline analyses. Subtle non-specific effects were observed in cre^(−/−)^ mice when laser powers are greater than 8mW (Baertsch et al., 2018). Therefore, as controls for our optogenetic photostimulation experiments, we used cre^(−/−)^ animals submitted to the same viral injections, optic fiber and EEG/EMG electrodes implants, and optogenetic stimulations as described above, and did not observe changes in any measured breathing parameters (N = 5, Fig. 1).

### Electrophysiology and astrocytes calcium imaging

#### Surgery

Twenty-one days following AAV-CAG-GCaMP injections into the VRC of Aldh1l1^Cre^ mice, respiratory parameters in all animals were recorded electrophysiologically and two-photon calcium imaging recordings were taken (Fig. 6A). Initially, general anesthesia was induced using urethane (1.5 g/kg, i.p., Sigma Aldrich, USA). To access the ventral medullary surface (VMS), a tube was place in the trachea (24 G) allowing the animals to spontaneously breathe 100% O_2_ for the remainder of the surgery and part of the experimental protocol. Core temperature was maintained through a water heating system (PolyScience, USA) built into the surgical table. The neck muscles were removed and a basioccipital craniotomy was performed. Pial vessels in the ventral marginal layer were located at 1.0 mm lateral from the basilar artery and 0.4 mm rostral to the most rostral portion of the hypoglossal nerve. A cover glass (3 mm round and 0.13 mm thickness, Warner Instruments, Germany) was placed covering the VMS.

Bipolar electrodes were placed to record diaphragm muscle (Dia_EMG_) activity with a signal amplification of 10,000x. Anesthesia depth was continuously assessed by testing the absence of withdrawal response; a supplemental dose of 0.01 mL of urethane was given to maintain adequate anesthetic depth. All analog data (EMG activity) were recorded via a Micro1401 digitizer (Cambridge Electronics Design, UK) and processed with Spike2 software (version 6). Integrated electromyography activity (〿EMG) was obtained through rectification, data was further processed using a band pass filtered (200–700Hz, 40Hz transition gap) and smoothing (σ = 0.002 s). DiaEMG was determined by averaging 〿EMG over 30 seconds, then normalized by assigning a baseline value recorded at room air. Raw data and differences (Δ) between baseline (normoxia) and hypoxia (10% O_2_) were reported.

#### Two-photon imaging

Imaging was performed on a Bruker Investigator microscope with Prairie-View imaging software, coupled to a Spectra-Physics InSight X3 tunable laser (680–1300 nm). A laser wavelength was set to 920 nm to excite GCaMP fluorescence, and the emitted signals were collected through 525/70 nm. A 10x (0.30 numerical aperture, NA) air objective (FN26.5, Olympus; UPLFLN10X2) was first used to navigate in medulla. We zoomed into the GCaMP expressing spots in VMS and acquire imaging data at 0.226 um/pixel resolution. Sequential stacks of images over two minutes each trial was acquired from the VMS to a depth of 100–300 mm.

#### Electrophysiology and Two-Photon Cross-Correlation Analyses

Each round of recordings consisted of ROIs generated at 600 time points over 4 minutes (~ 0.4-second intervals). The average Dia amplitude was calculated (~ 0.6 mV), and we defined a sigh as an inspiratory peak that exceeded twice the amplitude of an eupneic breath. We plotted each ROI vs. the Dia EMG amplitude for each recorded cell and correlated the unitary ROI with the time difference between the peak of each cell activity and the sigh generation. During hypoxia, we measured the multiunitary ROI of the region and correlated it with the time difference between population peak activity and peak Dia EMG.

We also plotted the ROI versus Dia EMG for eupneic activity, but in contrast to sighs, no correlation was found (data not shown).

### Histology

At the end of the experiments, all animals were deeply anesthetized with 4% isoflurane in 96% oxygen. They were then perfused through the ascending aorta with 20 ml of phosphate-buffered saline (PBS; pH 7.4), followed by 20 ml of 4% phosphate-buffered paraformaldehyde (0.1 M; pH 7.4; Electron Microscopy Sciences, Fort Washington, PA). The brains were removed and stored in the perfusion fixative at 4°C for 4 hours, then transferred to 20% sucrose for 8 hours. Coronal sections (25 μm) were cut from the brains using a cryostat and stored in a cryoprotectant solution at −20°C (20% glycerol plus 30% ethylene glycol in 50 ml phosphate buffer, pH 7.4) prior to histological processing. All histochemical procedures were performed using free-floating sections ^53^.

#### Immunohistochemistry:

ChAT was detected using a polyclonal goat anti-ChAT antibody (AB144P; Millipore; 1:100), eYFP was detected using a polyclonal chicken anti-GFP (1020; Ave Labs; 1:500), TH was detected using a polyclonal rabbit anti-TH (AB152; Millipore; 1:1000), GFAP was detected using a polyclonal rabbit anti-GFAP (Z0334; DAKO; 1:1,000), all diluted in PB containing 2% normal donkey serum (017–000-121, Jackson Immuno Research Laboratories) and 0.3% Triton X-100, and sections were incubated in it for 24 hours. The sections were then rinsed in PBS and incubated for 2 hours with secondary antibodies: Alexa 488 goat anti-chicken (A11039; Invitrogen; 1:500), Alexa 647 donkey anti-rabbit (A31573; Invitrogen; 1:500) and Alexa 647 donkey anti-goat (A21447; Invitrogen; 1:500). Control experiments confirmed that no labeling occurred when primary antibodies were omitted. Some sections were also stained with DAPI for 1 minute, rinsed with PBS, mounted on slides in rostrocaudal sequential order, dried, and covered with Fluoromount (00–4958-02; Thermo Fisher). Coverslips were affixed with nail polish.

Sections were also examined to confirm that fiber optics were placed in the intended location. Mice with incorrectly positioned fiber optics were excluded from the analysis. According to the Paxinos and Franklin (2012) mouse atlas ^55^, for targeting the preBötC, the unilateral tips were located just dorsal to the nucleus ambiguus near Bregma level − 6.84 mm, about 1 mm from the midline, and about 400 μm above the marginal layer (Fig. 1). For the RTN, the fiber tips were near the facial nucleus at Bregma level − 6.64 mm, about 1 mm from the midline, and about 250 μm above the marginal layer (Fig. 2).

#### In situ hybridization:

We utilized the RNAscope^™^ Multiplex Fluorescent Reagent kit (Advanced Cell Diagnostics). Initially, brain slices were incubated in 0.1M PBS for 5 minutes, followed by a 10-minute incubation with RNAscope Hydrogen Peroxide (#322381) at room temperature (RT). This was succeeded by three washes with PBS, each lasting 2 minutes. The tissue was then treated with Protease IV (#322381) for 30 minutes at RT, followed by another 2-minute incubation in 0.1M PBS. Next, the tissue was incubated with Aldh1l1 and Fos probes for 2 hours at 40°C, followed by a 2-minute wash with PBS. For hybridization, the tissue underwent sequential incubations with RNAscope Multiplex Detection Reagents (#323110) consisting of FL v2 Amp 1, for 30 minutes at 40°C, with a 5-minute PBS wash after each step. The tissue was then incubated with RNAscope Multiplex FL for 30 minutes at 40°C, followed by a 5-minute PBS wash. To develop the HRP-C1 signal and HRP-C2 signal, the tissue was incubated with RNAscope Multiplex FL v2 HRP-C1 or HRP-C2 for 15 minutes at 40°C, followed by a 5-minute wash with PBS. Finally, the tissue was treated with TSA Plus fluorescein (FP1168; amplification reagent, 1:250) for 30 minutes at 40°C, followed by a 5-minute PBS wash. The tissue was then incubated with RNAscope Multiplex FL v2 HRP blocker for 15 minutes at 40°C, followed by a 2-minute wash with PBS. All slides were mounted with Fluoromount (00–4958-02; Thermo Fisher), and coverslips were secured using nail polish.

### Cell Counting, Imaging, and Data Analysis

A VS120-S6-W Virtual Slide Scanner (Olympus) was used to scan all the sections. To avoid bias, photomicrography and counting were performed by a single researcher who was blinded. ImageJ (version 1.41; National Institutes of Health, Bethesda, MD) was used for cell counting or area measurements, and Canvas software (ACD Systems, Victoria, Canada, version 9.0) was used for line drawings. A one-in-two series of 25-μm brain sections was used per mouse, meaning that each section analyzed was 50 μm apart. We reported the analyzes as mean ± SEM. Section alignment for the RTN, preBötC, Nucleus of the solitary tract (NTS), *Locus coeruleus* (LC) and Kölliker-fuse/Parabrachial complex (KF/PB) was based on a reference section, following previous studies from our laboratory and the Paxinos and Franklin (2012) mouse atlas ^55^.

### Statistics

Statistical analysis was performed using Sigma Stat version 11.0 software (Jandel Corporation, Point Richmond, CA). Statistical tests included t-tests with follow-up Mann-Whitney U tests, one-way analysis of variance (ANOVA) with Dunn’s comparison test, or two-way ANOVA with Bonferroni’s multiple comparison test, as appropriate. Data are reported as mean ± standard error of the mean (SEM). A p-value of < 0.05 was considered statistically significant for all comparisons.

## Figures and Tables

**Table 1:** Ventilatory parameters of Aldh1l1 astrocytes opto-stimulation in VRC. f_R_ (breaths/min) and V_T_(μl/g).

Cre ^(−/−)^ VRC		Before	Light	Post-light	P	F
	Wake	248.2 ± 7.8	253.9 ± 6.6	256.8 ± 4.2	0.601	0.544
f_R_	NREMS	256.4 ± 9.7	251.5 ± 2.2	244.6 ± 4.8	0.453	0.877
	REMS	254.2 ± 5.1	243.8 ± 4.7	237.4 ± 5.7	0.101	0.517
	Wake	4.27 ± 0.09	4.36 ± 0.12	4.47 ± 0.08	0.463	0.850
V_T_	NREMS	4.94 ± 0.16	4.81 ± 0.10	4.71 ± 0.08	0.445	0.897
	REMS	4.85 ± 0.15	4.67 ± 0.11	4.88 ± 0.13	0.290	0.618
**Aldh1l1^Cre^**						
	Wake	262.7 ± 11.3	269.7 ± 7.9	262.8 ± 5.2	0.948	0.448
f_R_	NREMS	250.1 ± 7.9	244.2 ± 2.1	245.6 ± 4.8	0.645	0.455
	REMS	240.6 ± 3.6	242.5 ± 3.4	235.4 ± 5.0	0.364	1.101
	Wake	4.48 ± 0.15	**6.60 ± 0.27** *	**4.61 ± 0.07** ^#^	< 0.001	37.869
V_T_	NREMS	4.83 ± 0.10	**6.94 ± 0.29** *	**5.31 ± 0.25** ^#^	< 0.001	34.312
	REMS	4.90 ± 0.14	**6.12 ± 0.23** *	**4.92 ± 0.14** ^#^	< 0.001	64.658
**Aldh1l1^Cre^ + 6OHDA (A1/C1)**						
	Wake	238.0 ± 6.9	258.9 ± 9.0	255.7 ± 9.4	0.314	1.344
f_R_	NREMS	228.3 ± 5.6	268.5 ± 12.6	244.8 ± 11.8	0.109	2.954
	REMS	231.8 ± 11.7	270.8 ± 8.0	240.6 ± 12.7	0.089	3.328
	Wake	4.32 ± 0.09	**5.40 ± 0.15** *	**4.26 ± 0.05** ^#^	< 0.001	31.874
V_T_	NREMS	4.22 ± 0.09	**5.48 ± 0.15** *	**4.25 ± 0.15** ^#^	< 0.001	53.766
	REMS	4.34 ± 0.08	**5.67 ± 0.20** *	**4.07 ± 0.14** ^#^	< 0.001	28.733

* Compared to Before and

# Compared to Light (One-way repeated measures ANOVA)

**Table 2:** Percentage of variation promoted by Aldh1l1 astrocytes opto-stimulation in VRC compared to before stimulation.

		Cre ^(−/−)^	Aldh1l1^Cre^	Aldh1l1^Cre^ + 6OHDA (A1/C1)	P	F
Δ ^f^R (%)	Wake	2.85 ± 4.83	3.03 ± 1.89	9.43 ± 6.06	0.482	0.768
	NREMS	−1.35 ± 3.56	−1.87 ± 2.65	17.69 ± 6.14	0.064	5.513
	REMS	−3.96 ± 2.49	0.92 ± 2.03	**17.34 ± 3.06** * ^#^	**< 0.001**	**18.347**
	Wake	2.20 ± 2.80	**48.36 ± 8.36** *	**25.34 ± 5.09** * ^#^	**< 0.001**	**12.147**
ΔV_T_ (%)	NREMS	−2.13 ± 3.91	**44.24 ± 7.52** *	**29.97 ± 3.98** *	**< 0.001**	**14.860**
	REMS	−3.64 ± 1.30	**24.90 ± 2.95** *	**31.09 ± 6.03** *	**< 0.001**	**21.927**

* Compared to Cre ^(−/−)^ and

# Compared to Aldh1l1^Cre^ (One-way ANOVA)
